# Dr. Clarke's General Remarks, &c.

**Published:** 1813-04

**Authors:** 


### GENERAL REMARKS.

The lowest temperature in both the Tables is ascertained by a register thermometer on Six's construction, which presents the lowest degree of cold since the last observations, and always refers to a period of 24 hours; the greatest cold is generally admitted to be about one hour before sunrise. From an accurate account lately kept of the temperature at 11 at night, it has not been possible hitherto to obtain any rule for comparing these observations, the thermometer falling so irregularly in the course of the night; and in three instances last month (January) as much as 12°, although the difference in the mean of these observations for that month was only 4°. It is highly necessary this should be perfectly understood before any conclusions are drawn. The following Table of Temperatures, formed from observations made at different periods, may nevertheless be found interesting.

| 1812. | | *Strand, London. Noon, & 11 P. M.* | *4 Miles NNW of St. Paul's. Time not noted.* | *Plaistow. Time not noted.* | *Carlisle. Time not noted.* | *Bristol. Time not noted.* | *Derby, by a Register Thermometer.* | *Sidmouth, by a Register Thermometer.* |
|---|---|---|---|---|---|---|---|---|
| January . . | Highest | — | 45° | 50° | 50° | 48° | 47° | 49° |
| | Lowest | — | 22 | 26 | 10 | 20 | 25 | 20 |
| February . | Highest | 56° | 50 | 54 | 52 | 57 | 52 | 53 |
| | Lowest | 32 | 26 | 26 | 32 | 27 | 30 | 28 |
| March . . | Highest | 57 | 53 | 59 | 53 | 54 | 58 | 55 |
| | Lowest | 29 | 26 | 24 | 23 | 25 | 23 | 22 |
| April . . . | Highest | 56 | 57 | 58 | 51 | 56 | 55 | 57 |
| | Lowest | 28 | 24 | 25 | 30 | 35 | 26 | 26 |
| May . . . | Highest | 71 | 71 | 76 | 72 | 74 | 70 | 66 |
| | Lowest | 42 | 52 | 32 | 35 | 44 | 31 | 36 |
| June . . . | Highest | 74 | — | 75 | 76 | 73 | 72 | 71 |
| | Lowest | 48 | — | 39 | 46 | 45 | 34 | 37 |
| July . . . | Highest | 76 | — | 75 | 68 | 75 | 78 | 75 |
| | Lowest | 49 | — | 41 | 47 | 54 | 35 | 36 |
| August . . | Highest | 76 | — | 78 | 68 | 77 | 72 | 76 |
| | Lowest | 51 | — | 43 | 47 | 53 | 38 | 40 |
| September | Highest | 70 | — | 73 | 64 | 71 | 64 | 70 |
| | Lowest | 42 | — | 34 | 38 | 41 | 30 | 34 |
| October . | Highest | 66 | 65 | 69 | 60 | 65 | 59 | 65 |
| | Lowest | 34 | 40 | 34 | 33 | 31 | 27 | 31 |
| November | Highest | 54 | 54 | 55 | 53 | 52 | 49 | 57 |
| | Lowest | 26 | 26 | 24 | 20 | 17 | 23 | 24 |
| December | Highest | 53 | — | 52 | 48 | 52 | 44 | 54 |
| | Lowest | 23 | — | 18 | 15 | 14 | 19 | 23 |

### COMPARATIVE TEMPERATURES.

NOTTINGHAM.

1807. Highest temperature 80° July
1808. Highest ditto - - 89 July
Lowest ditto - - 17 Jan.
1809. Highest ditto - - 78 July
Lowest ditto - - 17 Jan.
1810. Highest ditto - - 82 Sept.
1810. Lowest temperature 14° Feb.
1811. Lowest ditto - - 13 Jan.

SIDMOUTH.

Mean for the 4 years 48.75
1811. Lowest temperature 22° Dec.
1812. Highest ditto - - 76 Aug.
Lowest ditto - - 20 Jan.

*Sidmouth, Feb.* 12, 1813.

